# Usefulness of Chromogenic Media with Fluconazole Supplementation for Presumptive Identification of *Candida auris*

**DOI:** 10.3390/diagnostics13020231

**Published:** 2023-01-08

**Authors:** Alba Ruiz-Gaitán, Ignacio Sigona-Giangreco, José Manuel Pérez-Royo, Victor Garcia-Bustos, Marta García-Hita, Eulogio Valentín-Gómez, Salvador Giner Almaraz, Piet W. J. de Groot, Javier Pemán

**Affiliations:** 1 Department of Clinical Microbiology, La Fe University and Polytechnic Hospital, 46026 Valencia, Spain; 2 Severe Infection Research Group, Medical Research Institute La Fe, 46026 Valencia, Spain; 3 Department of Internal Medicine, La Fe University and Polytechnic Hospital, 46026 Valencia, Spain; 4 Department of Microbiology and Ecology, University of Valencia, 46010 Valencia, Spain; 5 Regional Center for Biomedical Research, Castilla-La Mancha Science & Technology Park, University of Castilla-La Mancha, 02008 Albacete, Spain

**Keywords:** *Candida auris*, chromogenic media, CHROMagar™ Candida Plus, HiCrome^TM^ Candida, fluconazole supplementation, yeast identification

## Abstract

***Introduction*:***Candida auris* is a major threat to public health. Rapid detection is essential for early treatment and transmission control. The use of chromogenic media allows the presumptive identification of this new species. The aim of this study is to describe the morphological characteristics of *C. auris* colonies on three commercial chromogenic media. ***Methods***: Nineteen *C. auris* isolates from different countries/clades and 18 isolates of other species were cultivated in CHROMagar^TM^ Candida Plus, HiCrome^TM^ Candida, CHROMagar-Candida, and fluconazole-supplemented (32 mg/L) CHROMagar-Candida media. ***Results***: On CHROMagar^TM^ Candida Plus and HiCrome^TM^ Candida, *C. auris* isolates from Colombia, Venezuela, India, Korea, and Japan displayed blue-shaded colonies, while isolates from Spain and Germany exhibited light pink shades with a bluish halo. All isolates showed white to pink colonies on CHROMagar-Candida. On CHROMagar Candida supplemented with fluconazole, whilst *C. auris*, *C. glabrata*, or *C. krusei* showed a similar pink color at 48 h incubation, phenotypic differentiation was possible by the rough, paraffin-like texture or the intense purple color acquired by *C. krusei* and *C. glabrata*, respectively. Moreover, in this medium, the presence of *C. auris* in combination with other species of similar color was not limiting for its early identification, due to this medium selecting only strains resistant to this antifungal. ***Conclusions***: The use of chromogenic media such as CHROMagar^TM^ Candida Plus facilitates a presumptive identification of *C. auris*. However, this identification can be difficult in the presence of mixed cultures. In these cases, the use of CHROMagar^TM^ Candida medium with 32 mg/L fluconazole offers better performance for the identification of *C. auris* by inhibiting fluconazole-susceptible strains and selecting rare or high fluconazole MIC (>32 mg/L) isolates.

## 1. Introduction

Since its first description in 2009 [1], *Candida auris* has been responsible for nosocomial outbreaks in several countries around the world [2]. Distinguishing features of this species include its multi-resistance to antifungal drugs, its potential to cause deep-seated infections with elevated mortality rates [3], its high transmissibility and persistence in the hospital environment [3,4], and the difficulty of identification by conventional biochemical and phenotypic techniques. These characteristics have led the CDC to classify *C. auris* as a global public health threat [5,6].

Phenotypically, *C. auris* is difficult to distinguish from other *Candida* species. On Sabouraud agar, *C. auris* forms white to cream colonies, and on chromogenic media such as CHROMagar Candida, it forms light pink colonies [7,8].

Available commercial identification systems generally misidentify *C. auris* as many other species with greater or lesser phylogenetic closeness, such as *Candida haemulonii*, *Rhodotorula glutinis*, *Saccharomyces cerevisiae*, or, less frequently, *Candida famata*, *Candida dobushaemulonii*, *Candida sake*, *Candida lusitaniae*, *Candida albicans*, *Candida guilliermondii*, or *Candida parapsilosis* [5].

Since the characterization of this yeast as an emerging global threat to public health and the acknowledgment of the difficulties for its identification using conventional biochemical techniques, such as API ID20C (bioMérieux) and AuxaColor™ 2 (BioRad-Laboratories, Marnes-la-Coquette, France), efforts have been driven to develop rapid detection methods with advanced techniques capable of avoiding the limitations of phenotypic tests, such as mass spectrometry (MALDI-TOF MS), or molecular techniques such as internal transcribed spacer sequencing (ITS). Despite their accuracy, these techniques are expensive and require qualified personnel, hampering their use at non-specialized centers or especially resource-limited settings. This fact limits not only the early initiation of appropriate treatment, but also the early detection of cases and contacts [9,10]. Furthermore, all suspected cases of *C. auris* colonization or infection should be immediately isolated owing to its high transmissibility in the hospital environment and contact cases must be promptly identified and screened for *C. auris.* In this matter, it is essential to avoid the delay in the microbiological identification of *C. auris*, limit or control the generation of hospital outbreaks, and diminish patient morbimortality as well as the healthcare-associated burden [11]. The still-present lack of knowledge on easily reproducible, low-cost but sensitive techniques for prompt *C. auris* identification widens the gap in equity between developing countries [12].

Most microbiology laboratories perform presumptive identification of *Candida* spp. using chromogenic media [13,14]. Previous reports on CHROMagar^TM^ *Candida* medium have shown that it is not useful for differentially identifying *C. auris* and *C. parapsilosis* colonies [15]. Consequently, other selective media such as CHROMagar^TM^ Candida Plus medium (CHROMagar, Paris, France) and HiCrome^TM^ Candida have been developed to further discriminate *C. auris* colonies from other species [16,17,18]. Despite recent progress in this area, microbiological data with these modified media for presumptive identification of *C. auris* are still limited to studies with a relatively low number of species and strains tested, discrepancies between morphotypes, or lack of information on mixed cultures.

Therefore, the aim of this study was to describe the phenotypic characteristic of *C. auris* colonies on four commercially available chromogenic media, as well as to evaluate their performance for presumptive identification of *C. auris* compared to other non-*C. auris* species and mixed cultures, including a modified fluconazole-supplemented CHROMagar^TM^ Candida.

## 2. Materials and Methods

A total of 35 suspensions of pathogenic yeast species were inoculated in CHROMagar^TM^ Candida Plus (CHROMagar, Paris, France), HiCrome^TM^ Candida (HIMEDIA LBS, Mumbai, India), CHROMagar^TM^- Candida (Biolife, Milan, Italy), and CHROMagar Candida supplemented with 32 mg/L of fluconazole (CHROMagar Candida plus fluconazole) (MAIM, Barcelona, Spain). Of these, 19 *C. auris* strains from different countries were selected to represent the four main global clonal lineages. Thirteen of these strains were from the Westerdijk Fungal Biodiversity Institute (CBS-KNAW): CBS-12766, CBS-12882, CBS-12766 (clade I; India); CBS-12372, CBS-12373 (clade II; Korea); CBS-10913, JM-15448 (clade II: Japan); CBS-15603, CBS-15604, CBS-15605, CBS-15606, CBS-15607, CBS-15610 (clade III: Spain); COL-01, COL-02, COL-03 (clade IV: Colombia), 10-05-15-23, 10-05-15-19, 10-05-15-22 (clade IV: Venezuela). Also, 5 reference *Candida* strains were analyzed (*C. albicans* ATCC-90028, *Candida glabrata* ATCC-90030, *Candida tropicalis ATCC-200956, C. lusitaniae* ATCC-135, and *Candida krusei* ATCC-6258), as well as one each of *Candida utilis, Candida rugosa, Candida orthopsilosis, Candida metapsilosis, Candida boidinii, Candida nivariensis, C. guillermondii, S. cerevisiae, Geotrichum capitatum, Trichosporon asahii, Kodamaea ohmeri,* and *Rodothorula glutinis* from clinical samples. Identification of all strains were confirmed by MALDI-TOF VITEK-MS^®^ (bioMérieux, Marcy-l`Étoile, France) and sequencing of ITS 1-2 [19].

For monocultures, inoculum suspensions were prepared at 0.5 McFarland, adjusted to a concentration of 10^4^ colony-forming units (CFU), and inoculated on three chromogenic media using a calibrated 5 µL pipette. All strains in monoculture were incubated at 37 °C for 48 h. Four mixed cultures containing a *C. auris* strain with 3 other non-*C. auris* species were performed to assess their ability to differentiate among *Candida* species. For all mixed cultures, inoculum suspensions were prepared at 0.5 McFarland and adjusted to 10^4^ CFU. Then, the inoculum was adjusted to 10^3^ CFU, and suspensions were inoculated on four chromogenic media using a calibrated 100 µL pipette. Mixed cultures were incubated at 37 °C and read at 24 h and 48 h by a clinical mycologist. Recording of color, size, and texture of colonies in each medium was performed both visually and using a magnifying lens (Nikon SMZ1500).

## 3. Results

**Figure 1:**
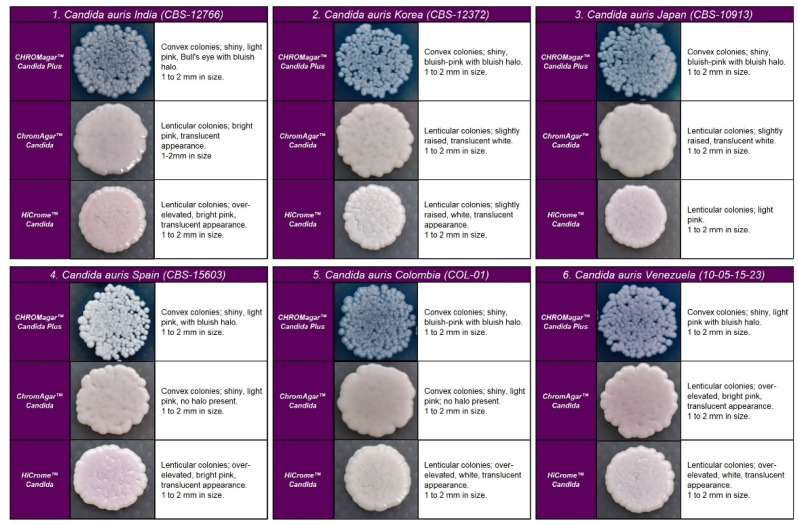
Appearance of *Candida auris* colonies of the four clades described on CHROMagar™ Candida Plus medium, CHROMagar™ Candida medium, and HiCrome^TM^ Candida after 48 h incubation at 35 °C.

In the four chromogenic media analyzed, the most specific morphology was observed at 48 h in all strains (Figure 1). Recovered colonies were presumptively identified according to the colony color described by the manufacturer.

*C. auris* strains developed similar colonies in size, color, and texture in each medium. In CHROMagar^TM^ Candida Plus and HiCrome^TM^ Candida, *C. auris* strains from Colombia, Venezuela, India, Korea, and Japan exhibited blue-shaded colonies, while strains from Spain exhibited light pink with a bluish halo that diffuses into the surrounding agar. By analysis using HiCrome^TM^ Candida, *C. auris* showed pale pink shades without a halo (see Figure 1).

**Figure 2:**
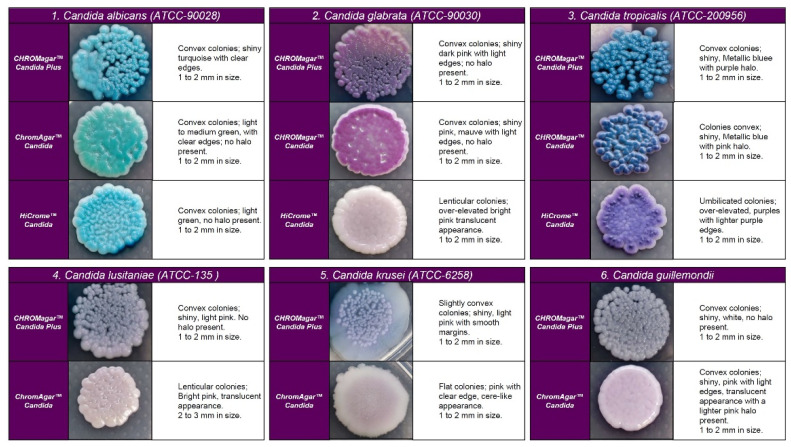
Appearance of colonies of most pathogenic *Candida* species on CHROMagar™ Candida Plus medium, CHROMagar™ Candida medium, and HiCrome^TM^ Candida after 48 h incubation at 35 °C.

On CHROMagar^TM^-Candida, all *C. auris* strains showed white and pink colonies at 24 and 48 h, respectively. Colonies of *C. auris, C. glabrata,* or *C. krusei* on CHROMagar Candida plus fluconazole showed a similar pink color after 48 h incubation; however, phenotypic differentiation was possible by the rough, paraffin-like texture or the intense purple color acquired by *C. krusei* and *C. glabrata*, respectively ( Figure 1 and Figure 2).

**Figure 3:**
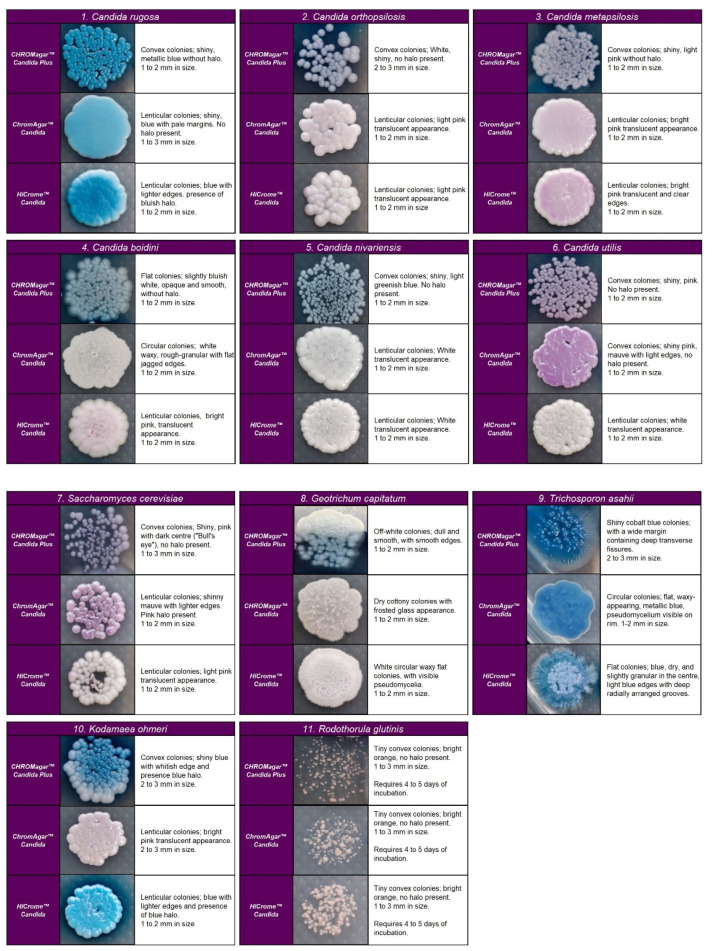
Colony appearance of five non-*Candida species* on CHROMagar™ Candida Plus, CHROMagar™ Candida, and HiCrome^TM^ Candida after 48 h incubation at 35 °C.

After 24 h incubation on CHROMagar Candida plus fluconazole, *C. auris* showed smooth, convex, shiny, and light pink colonies (colony size 0.7–3 mm). After 48 h, they acquired an egg yolk morphology (white periphery and purple center). Although *C. auris, C. glabrata*, or *C. krusei* showed a similar pink color at 48 h incubation, phenotypic differentiation was possible due to the same characteristics observed for *C. krusei* and *C. glabrata* on CHROMagar^TM^-Candida. All *C. auris* strains but COL-01 and CBS-10913 were able to grow (these two isolates were susceptible to fluconazole), while *S. cerevisiae*, *C. albicans*, and *C. famata* showed inhibition of growth in this fluconazole-supplemented medium ( Figure 2 and Figure 3).

Regarding the colony morphology, the strains grown on CHROMagar^TM^ Candida Plus developed individualized colonies, in contrast to the other three media where the growth is agglomerated at 48 h ( Figure 2 and Figure 3). On CHROMagar^TM^ Candida and HiCrome^TM^ Candida, after this incubation time, differentiation between the mauve/light pink species (*C. auris*, *C. ortopsilosis*, *C. metapsilosis*, *C. nivariensis*, *C. guillermondii*, and *C. lusitaniae*) was difficult. In CHROMagar^TM^ Candida Plus, some species (*C. albicans*, *C. tropicalis*, *C. rugose,* and *K. ohmeri*) developed a similar metallic blue color, the distinction of which was difficult without significant changes with incubations over 48 h (Figure 2). Similar results were reported for species such as *C. auris*, *C. albicans*, *C. parapsilosis/orthopsilosis*, or *C. tropicalis* that presented a bluish color. However, differentiation was possible through the variety of shades or halos with different colors [17].

**Figure 4:**
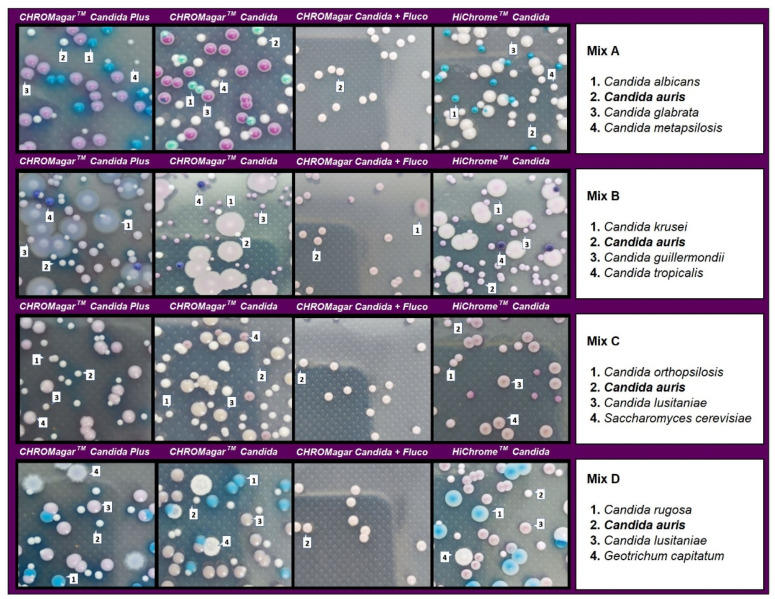
Appearance of *Candida species* and other yeast species colonies from mixed cultures on CHROMagar™ Candida Plus, CHROMagar™ Candida, CHROMagar™ Candida plus fluconazole, and HiCrome^TM^ Candida after 48 h incubation at 35 °C.

In the evaluation of mixed cultures, on CHROMagar Candida plus fluconazole, *C. auris* showed smooth, convex, shiny, light purple colonies with an egg yolk morphology (white periphery and purple center). After 48 h of incubation, in cultures including *C. auris* in combination with *C. albicans*, *C. metapsilosis,* and *C. glabrata*, identification was difficult in the three tested media due to the whitish to pinkish coloration of the colonies. However, after 48 h incubation, *C. auris* colonies acquired a turquoise blue halo in CHROMagar^TM^ Candida Plus that encouraged differentiation. All *C. albicans*, *C. metapsilosis*, and *C. glabrata* isolates were completely inhibited on this medium (Figure 4).

In mixed cultures, the number of colonies was similar for CHROMagar^TM^ Candida Plus and CHROMagar^TM^ Candida with some differences depending on the species assayed. CHROMagar Candida plus fluconazole was able to recover 97–100% of *C. auris* colonies before 48 h of incubation. Differentiation was not possible with HiCrome^TM^ Candida in mixed cultures containing *C. auris* and *C. guillermondii* (Table 1).

## 4. Discussion

Rapid and reliable diagnosis of *C. auris* infection or colonization is essential for prompt and accurate antifungal therapy as well as for transmission control. Classical yeast identification techniques still available such as API-20C, AuxaColor^TM^ 2, VITEK-2 YST, BD-Phoenix^TM^, and MicroScan misidentify *C. auris* [20,21]. To avoid the identification problems of available commercial techniques, the use of commercial media such as CHROMagar Candida^®^ supplemented with Pal agar (sunflower seed extract) has been suggested as a simple and low-cost method to distinguish between *C. auris* isolates and the *C. haemulonii* complex [22].

New media such as HiCrome^TM^ Candida and CHROMagar^TM^ Candida Plus have been previously studied with identification rates between 0% and 33%, respectively [21]. Our results using CHROMagar^TM^ Candida Plus agree with those described by other authors who reported good results in the detection of *C. auris* in both artificial cultures and surveillance samples after 36 h of incubation, respectively [17,21]. Identification of *C. auris* was difficult in the presence of white to mauve growing species at 24–36 h of incubation [17]. In our study, after 24 h of incubation, inhibition of susceptible species in CHROMagar^TM^ Candida supplemented with 32 mg/L fluconazole allows a rapid recovery of *C. auris* colonies, although those were small and not colored enough.

In studies carried out with CHROMagar^TM^ Candida Plus, it has been described that *C. auris, C. albicans, C. parapsilosis/orthopsilosis,* or *C. tropicalis* species can have a similar bluish color before 36 h [17,21,23]; in our study, we found similar results for *C. auris, C. orthopsilosis, C. metapsilosis, C. nivariensis,* and *C. glabrata* in this medium.

According to our results, in culture assays performed on CHROMagar Candida with 32 mg/L of fluconazole, the presence of *C. auris* in combination with other species of similar color was not limiting for its early identification, due to this medium selecting only fluconazole-resistant strains. Although *C. auris* isolation in this medium requires further confirmation, it allows for a prompt response that enables rapid isolation of patients and thus improved control of its transmission.

Currently, molecular methods or mass spectrophotometry (MALDI-TOF MS) are recommended for the definitive identification of *C. auris*. Among the molecular methods, sequencing of genetic loci (D1/D2, RPB1 and RPB2), rRNA internal transcribed spacers (ITS), and commercialized molecular technologies are the most commonly used [24,25]. These techniques are reliable but their availability outside reference laboratories is limited, especially in developing countries [26,27]. In these settings, the use of CHROMagar^TM^ Candida agar with 32 mg/L of fluconazole as the primary culture medium in patients with suspected *C. auris* colonization allows fast presumptive identification of *C. auris* (24–48 h). This achievement is similar to that observed by other authors who used CHROMagar^TM^ Candida Plus for the isolation and identification of *C. auris* from clinical samples in monoculture [17,21]. Although a presumptive identification can be achieved in 36 h, in all the media studied, a minimum incubation of 48 h is necessary to facilitate the differentiation of the species, and definitive identification must be done by spectrophotometric techniques such as MALDI-TOF or by molecular methodology sequencing the ITS regions [28,29,30].

In conclusion, the use of low-cost and accessible chromogenic media could allow the presumptive identification of *C. auris* infection or colonization in areas with a high prevalence of this species. This advantage is more evident with the use of CHROMagar^TM^ Candida agar with 32 mg/L of fluconazole: this medium offers better performance for the identification of *C. auris* by inhibiting other species susceptible to this antifungal and selecting rare strains or those with high fluconazole MIC (>32 mg/L). Nevertheless, in all cases, definitive confirmation is required.

## Figures and Tables

**Table 1:** Comparison of CHROMagar^TM^ Candida Plus, HiCrome^TM^ Candida, CHROMagar^TM^ Candida, and CHROMagar Candida supplemented with 32 mg/L of fluconazole for the recovery (CFU) of mixed cultures after 48 h of incubation at 35 °C.

Mixed Culture/Organism	Recovered Colonies (n)			
	CHROMagar^TM^ Candida Plus	HiCrome^TM^ Candida	CHROMagar^TM^ Candida	CHROMagar Candida Plus Fluconazole
**Mix A**				
*C. auris*	142	74	144	112
*C. glabrata*	137	134	137	NG
*C. metapsilosis*	32	85	30	NG
*C. albicans*	65	65	74	NG
Total	376	358	385	112
*C. auris* recovered vs. total (%)	37.77	20.67	37.40	100
**Mix B**				
*C. auris*	100	* 325	81	124
*C. guillermondii*	213		166	NG
*C. tropicalis*	21	37	13	NG
*C. krusei*	89	77	67	3
Total	423	114	327	127
*C. auris* recovered vs. total (%)	23.64	ND	24.77	97.64
**Mix C**				
*C. auris*	105	122	107	103
*C. lusitaniae*	108	107	108	NG
*S. cerevisiae*	39	35	28	NG
*C. orthopsilosis*	85	20	70	NG
Total	337	284	313	103
*C. auris* recovered vs. total (%)	31.16	42.96	34.19	100
**Mix D**				
*C. auris*	105	85	72	74
*C. rugosa*	57	57	35	NG
*C. lusitaniae*	96	49	130	NG
*G. capitatum*	37	24	19	NG
Total	295	215	256	74
*C. auris* recovered vs. total (%)	35.59	39.53	28.13	100

NG = Non-growth; ND = not determined; * uncountable.

## Data Availability

Not applicable.
